# Preliminary Data on the Occurrence of *Anisakis* spp. in European Hake (*Merluccius merluccius*) Caught Off the Portuguese Coast and on Reports of Human Anisakiosis in Portugal

**DOI:** 10.3390/microorganisms10020331

**Published:** 2022-02-01

**Authors:** Maria J. Santos, Matilde Matos, Lisa Guardone, Olwen Golden, Andrea Armani, Andreia J. R. Caldeira, Madalena Vieira-Pinto

**Affiliations:** 1 Laboratório de Patologia Animal, Departamento de Biologia, Faculdade de Ciências da Universidade do Porto, Rua do Campo Alegre s/n, FC4, 4169-007 Porto, Portugal; 2 Laboratório de Patologia Animal, CIIMAR—Centro Interdisciplinar de Investigação Marinha e Ambiental, Universidade do Porto, Terminal de Cruzeiros do Porto de Leixões, Av. General Norton de Matos s/n, 4450-208 Matosinhos, Portugal; matilde.matos30@gmail.com (M.M.); olwengolden@gmail.com (O.G.); profaandreiajuliana@gmail.com (A.J.R.C.); 3 Departamento de Ciências Veterinárias, Universidade de Trás-os-Montes e Alto Douro, Quinta de Prados, 5001-801 Vila Real, Portugal; mmvpinto@utad.pt; 4 FishLab, Department of Veterinary Sciences, University of Pisa, Viale delle Piagge 2, 56124 Pisa, Italy; lisa.guardone@for.unipi.it (L.G.); andrea.armani@unipi.it (A.A.); 5 Academic Institute of Health and Biological Sciences, Goias State University, Campus Central–Sede Anapolis (CET), BR 153, CEP 75132-903 Goias, Brazil; 6 CECAV, Centro de Ciência Animal e Veterinária, Universidade de Trás-os-Montes e Alto Douro, Quinta de Prados, 5000-801 Vila Real, Portugal

**Keywords:** *Anisakis simplex*, *Anisakis pegreffii*, food safety, risk analysis, one health

## Abstract

Parasitic nematodes of the genus *Anisakis* are among the most important biological hazards associated with seafood. A survey of *Anisakis* spp. in European hake (*Merluccius merluccius*) was undertaken as this species is a staple of the Portuguese diet. Moreover, a literature review of cases of anisakiosis reported from Portugal, a country with one of the highest levels of fish consumption in the world, was also carried out. Seventy-five European hake caught in the Atlantic Ocean off the northern coast of Portugal were analyzed to determine the infection levels and site distribution of *Anisakis* spp. Isolated nematode larvae were identified to species level by molecular analysis. Two sets of samples were collected. Firstly, a total of 46 *Anisakis* spp. L_3_ larvae were collected with a prevalence of 76.7% (95% CI 61.5–91.8%) and intensity (mean ± SD, range) of 2.0 ± 1.2 (1–5). Most larvae were found on the liver (45.7%) and on the gonads (32.6%), but none in the muscle. The molecular analysis showed the presence of both *A. simplex* s.s. (70%) and *A. pegreffii* (30%). For the second sample, analyzed using the UV-Press method, a total of 473 *Anisakis* spp. were found, with a prevalence of 95.6% (95% CI 89.5–100.0%), intensity (mean ± SD, range) of 11.3 ± 9.7 (1–41), density of 0.05 ± 0.04 (0–0.16) worms/muscle weight in g, and density of 0.54 ± 0.50 (0–2.53) worms/viscera weight in g. Surprisingly, only three very recent cases of human anisakiosis in Portugal have been reported in the literature. Data from this study contribute towards an updating of the existing epidemiological picture in an area characterized by very high seafood consumption and changing eating habits.

## 1. Introduction

The genus *Anisakis* has a global distribution and an indirect life cycle. Cetaceans and other marine mammals are definitive hosts, whereas crustaceans (Euphausiacea) act as first intermediate hosts. The latter may be eaten by the definitive host and the second intermediate host, which can be fish or cephalopods, also called paratenic hosts, where *Anisakis* spp. third stage larvae (L_3_) can be found in the viscera and, less frequently, in the muscle [1]. Humans can become accidental hosts after the ingestion of infected raw fish or cephalopods. The genus *Anisakis* has nine molecularly validated species: *A. simplex* and *A. pegreffii* are the most common and are linked to human infections and, along with *A. berlandi, A. ziphidarum, A. nascettii,* and *A. typica,* belong to larval morphotype I; the species *A. physeteris* belongs to larval morphotype II; *A. brevispiculata* to larval morphotype III; and *A. paggiae* to larval morphotype type IV [1]. These morphotypes are distinguished by the presence or absence of a mucron, the shape of the ventricle, tail shape, and their dimensions [1]. For many years, only two larval morphotypes were recognized, types I and II [1].

Human infection by nematodes of the family Anisakidae, and in particular *Anisakis* spp. [2]., is known as anisakiosis [3], although the term anisakiasis is also commonly used [1]. The typical presentation involves acute or chronic gastrointestinal symptoms [4], but mild to severe allergic reactions may also occur [5,6,7,8]. Although the allergenic potential of dead larvae is still debated [6,9], there is a wide consensus that, after sensitization with live larvae, exposure to the allergens of dead parasites is sufficient to elicit an allergic response [2]. Over the past 30 years, a marked increase in the prevalence of anisakiosis has been reported throughout the world, which may be due to the improvement of diagnostic techniques, greater knowledge of the parasite, and the increasing popularity of raw or lightly cooked seafood [2,10,11]

Anisakiosis can manifest itself as several types of disease, such as gastric anisakiosis, intestinal anisakiosis, ectopic anisakiosis, and gastroallergic anisakiosis. The first two types occur after the ingestion of raw fish when the larvae penetrate the stomach or the intestine, respectively. With both of these types of disease, symptoms of sudden abdominal pain for a few hours to several days may occur, as well as inflammatory reactions, eventually leading to granuloma formation. The third type, ectopic anisakiosis, involves the penetration of larvae into other tissues, such as the pharynx, tongue, lung, peritoneal cavity, lymphatic ganglia, or pancreas, and also occurs after the ingestion of raw fish. The fourth type, gastroallergic anisakiosis, manifests itself in the form of acute allergic symptoms, ranging from urticaria or angioedema to anaphylaxis, and the larvae ingested are usually expelled by the allergic reaction itself (vomiting and diarrhea), and thus they do not need to be extracted via gastroscopy, as they do for the first two types. The latter type can often occur as occupational allergic anisakiosis, in which the ingestion of larvae, or an oral exposure, is not required, and sensitization occurs via *Anisakis* spp. proteins coming into contact with the skin or respiratory tract. This is very common among individuals that deal with fish, such as fishery or aquaculture workers, chefs, fishmongers, and anglers [12,13].

Portugal, like Spain, has one the highest rates of fish consumption in the world (56.8 kg per capita per year in 2017) [14]. Spanish people frequently consume undercooked seafood: the most common source of infection is traditionally marinated anchovies [15], but cases due to hake consumption have also been reported [16,17]. The Portuguese, on the contrary, are more accustomed to eating cooked fish [18] as the traditional diet does not include raw seafood dishes [19,20]. However, culinary habits are changing, and uncooked or lightly cooked seafood is increasingly popular.

Among several other fish species, the European hake (*Merluccius* (Linnaeus, 1758) may host anisakid larvae. It is a common catch in the North East Atlantic and Mediterranean and one of the most important demersal fish stocks in European waters [21,22]. The European hake is one of the most popular fish in Portugal, where not only the flesh but also the gonads, a common site of infection, are extensively used as food. Surprisingly, data on human anisakiosis in Portugal appear to be scarce.

In order to supplement existing epidemiological data in this country, the aim of the present study was: (1) to determine the infection levels and site distribution of *Anisakis* spp. L_3_ in European hake caught in the North East Atlantic close to the city of Matosinhos, northern Portugal, and (2) to conduct a brief review of human cases of anisakiosis in Portugal since no such review currently exists.

## 2. Materials and Methods

### 2.1. Fish Sampling and Parasitological Examination

Two sets of samples were collected, one in 2015 (*n* = 30 hakes) and another in 2021 (*n* = 45 hakes). Seventy-five specimens of European hake from the North East Atlantic were included, caught locally by inshore fishing boats near Matosinhos harbor off the northern coast of Portugal. In each sample, the fish were collected by randomly sampling 10 specimens from 3 different lots in September 2015, and 15 and 30 specimens from 2 different lots during April 2021. The number of fish in the second sample was higher because we wanted to repeat the analysis in juvenile fish (the main set of fish in the first sample) and extend it to a set of more developed fish in order to get a wider view of the hake population status.

Two stocks of hake are recognized in EU Atlantic waters: the northern one in the North Sea, Skagerrak, and off the Atlantic coasts of UK, Ireland and France, while the southern one occurs off the Atlantic coasts of Spain and Portugal [22]. The 75 specimens analyzed in this study came from the southern stock.

The fish were collected fresh, quickly transported to the laboratory on ice and frozen in individual plastic bags until examination. After defrosting, and before dissection, all fishes were measured (total length; TL) to the nearest 0.1 cm and weighed (±0.1 g) (Table 1). For the first sample, the body cavity and the viscera were visually inspected, as described in [23]. Muscle fillets from the epaxial and hypaxial region (including the belly flaps around the viscera) were carefully sliced with a knife, ensuring that the muscle tissue was as clean as possible. They were first subjected to compression and then to visual inspection, conducted as described below and following the European Union Reference Laboratory for Parasites protocol (Detection of Anisakidae L3 larvae in fish fillets, https://www.iss.it/documents/20126/2428738/MO_POPVI_04_03_Instruction_PT_Anisakis_rev_3_.pdf/63dd4c65-243c-d83b-c9c2-8d9a11fccef0?t=1575743532629 accessed at 9 January 2015). In detail, after the fillets were cut as thin as possible, they were examined by placing them between two strong sheets of plexiglass, and compression was applied to obtain slices 1–2 mm thick in order to allow light penetration through the tissue. A stereoscope was used with an up-light capability to carefully and slowly scan each sample for *Anisakis* larvae. Larvae were collected and isolated before being counted, washed by immersion in saline solution (0.9% NaCl), and subsequently preserved in 70% Ethanol. The worms were cleared in glycerin and mounted individually on slides for light microscopical observation in order to obtain the generic identification and distinction between Type I and other types of *Anisakis* L_3_ [24,25]. The larvae were also measured for various morphometric features, such as esophageal length and ventricular length and width, in order to separate the larvae of *A. simplex* from *A. pegreffii*, according to the method described by Quiazon et al. [26]. This identification was later repeated using molecular analysis.

In the second sample, the fish were prepared and examined according to the method described by Karl and Leineman in 1993 [27], i.e., the UV-Press method. Briefly, fish were thawed, measured, and weighed (Table 1). The viscera were removed, weighed and placed in a transparent plastic bag for compression. Muscle fillets from the epaxial and hypaxial region (including the belly flaps) were removed, skinned, and weighed. They were then placed in a bag for compression. For larger fish, the belly flaps were placed in a separate bag to the dorsal musculature. For small fish, all of the muscle was placed in one bag.

The bags of viscera and muscle were placed between two metal plates and squeezed to a thin layer of about 2 mm. The compressed samples were then frozen at −20 °C for a minimum of 12 h. The samples were thawed and examined using UV light at 366 nm in a darkened room. The fluorescent larvae were identified, and their location was marked on the plastic bag. A different method was used in the second sample, as we did not find any larvae in the muscle with the first method, and we were concerned that this was due to the detection method used. Moreover, we aimed to compare both methods using fish from the same locality.

Once identified, the larvae were removed from the samples, counted, washed in 0.9% saline solution and examined using a stereoscope to distinguish between Type I and other Types of *Anisakis* L_3_. The presence of a tail spine or mucron was used to identify Type I larvae.

The parasitic load was determined using the parameters: prevalence (%), intensity, abundance and density (mean ± SD, range) (see Table 1). Density was defined as the number of worms ≥0/g of muscle or the number of worms ≥0/g of viscera, following Bush et al. [28]. The distribution of worms in each organ was determined as a percentage for sample 1.

In the second sample of fish, 14 of the 45 fish analyzed were partly or fully eviscerated. In the 11 partly eviscerated fish, there was still sufficient viscera remaining to examine and many of these samples contained several larvae. Three fish were fully eviscerated, and these were excluded when calculating the visceral abundance and density of worms per gram of viscera.

The condition factor of the fish was calculated using the formula: Condition Factor (Krel) = W/aL^b from the formula Log W = Log a + bLog L (where W = weight, L = length, b = slope of the line, and log a = its position) [29] (Table 1).

A statistical analysis was conducted, analyzing different host or parasite variables with the data from the second sample. The fish were separated into two equal groups, according to their weight. The lighter group (with weights between 95 and 189 g) contained 23 fish, and the heavier (with weights between 208 and 427 g) 22 fish. The abundance per fish, thevisceral and muscle larval abundance, and the visceral and muscle density were compared between the two groups, using the Mann–Whitney U test. Moreover, some host–parasite correlations were evaluated using the Spearman correlation coefficient. Fish length, weight, and condition factor were correlated with fish abundance, viscera larval abundance, muscle larval abundance, visceral density, and muscle density. Visceral abundance was correlated with muscle abundance and visceral density with muscle density. The level of significance was set at *p* < 0.05 for all comparisons. The software used for the statistical analysis was SPSS, version 27.

### 2.2. Molecular Identification

Molecular identification was conducted on a subsample (35%) of the parasites found in the first sample. Extraction of total DNA was performed as described by Guardone et al. [30]. Quali-quantitive analysis of extracted DNA was conducted by a NanoDrop ND-1000 spectrophotometer (NanoDrop Technologies, Wilmington, DE, USA). A fragment (~600-bp) of the mitochondrial cytochrome c oxidase subunit II (cox2) gene was amplified using the primers n. 211 (5′-TTTTCTAGTTATATAGATTGRTTTYAT-3′) and n. 210 (5′-CACCAACTCTTAAAATTA TC-3′) from Nadler and Hudspeth [31]. In addition, a fragment of about 900-bp of the ITS-1 region, the 5.8S gene and the ITS-2 region plus approximately 70 nucleotides of the 28S gene (abbreviated below as ITS) was amplified according to Zhu et al. [32]. PCR products were analyzed by electrophoresis in 2% agarose gel, and those of the expected length were submitted to forward and reverse Sanger sequencing by an external company. The sequences obtained were analyzed using BioEdit software and compared with sequences deposited in GenBank.

### 2.3. Literature Analysis for Human Anisakiosis Cases in Portugal

A literature search of cases of human anisakiosis in Portugal was conducted on PubMed (using MeSH terms in order to account for variations in language), Google Scholar, and Web of Science databases, using the following keywords and was applied to all fields: (Anisakidae OR *Anisakis*) AND (human OR human cases) OR (anisakidosis OR anisakiasis OR anisakiosis) AND Portugal. The English terms were also translated into French, Italian, Portuguese, and Spanish to perform dedicated search on databases in order to retrieve articles in these languages. Moreover, some Portuguese databases were also searched as “sapo.pt” and “aeiou.pt” in order not to miss any national citations. The search was conducted using terms together or combined differently in order to retrieve the maximum number of records. The reference lists of the screened articles were also checked. No starting date time limit was applied to the search, which was concluded in December 2021.

## 3. Results and Discussion

### 3.1. Parasitological Examination of Hake Specimens

*Anisakis* spp. infection in the European hake has been investigated in several previous studies [21,22,33,34,35,36]. However, considering the very high consumption of seafood in the Portuguese diet and knowing that the European hake is part of the staple diet of the Portuguese population and one of the most important demersal species captured off Western Europe, additional data on the parasitic occurrence, infection intensity, location, and species identification are required to enhance existing epidemiological data.

The fish of the first sample had a mean ± SD length of 28.3 ± 2.1 cm (range 24.2–32.9 cm) and mean ± SD weight of 225.2 ± 44.4 g (range 122.1–288.2 g), whereas those of the second sample had a mean ± SD length of 31.6 ± 3.7 cm (range 25.0–38.0 cm) and a mean ± SD weight of 212.6 ± 85.7 g (range 95.0–427.0 g).

**Figure 1:**
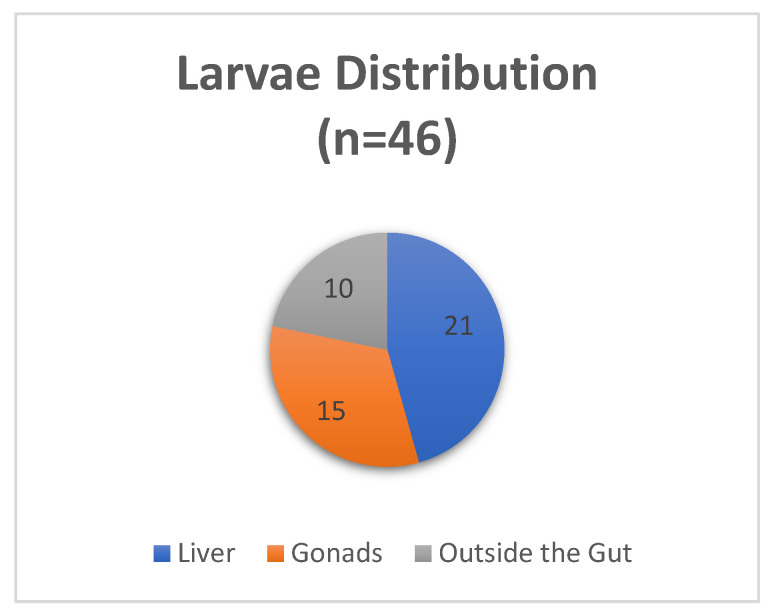
*Anisakis* spp. larvae distribution on fish viscera in the first sample.

In our first sample, a total of 46 *Anisakis* spp. L_3_ larvae were found, with a prevalence of 76.7% (95% CI 61.5–91.8%), mean intensity of 2.0 and mean abundance of 1.6 (see Table 2). All worms found were encapsulated and embedded in the surfaces of visceral organs, exhibiting a coiled shape. The majority of the larvae was found on the liver (*n* = 21 worms, 46% of the total L_3_ larvae) and on the gonads (*n* = 15 worms, 32%), whereas the remaining 10 larvae (22%) were located outside the gut (Figure 1). In the majority of samples (*n* = 18 fishes), there was no co-occurrence of the worms in the liver and in the gonads. Worms were present in both organs in only three fish. A higher percentage of parasite infection occurred on the liver (65%) and lower a value on the gonads (14%) was also reported for European hake from the Adriatic Sea [37]. No larvae were found in the muscle tissue of the specimens analyzed in this sample.

In our second sample, a total of 473 *Anisakis* spp. L_3_ larvae were found, with a prevalence of 95.6%, mean intensity of 11.0, and mean abundance of 10.5 (Table 2). The mean density of worms per gram of muscle was 0.05, and the mean density per gram of viscera was 0.54 (Table 2). Comparing the infection values of the two samples, it was apparent that the number of larvae detected by UV-Press method applied to the second sample was much more sensitive than that of the first sample. In particular, in detection of larvae in the muscle, we found no larvae using the first method but many larvae using the second method; thus, we conclude that the latter is more efficient at detecting these larvae.

**Figure 2:**
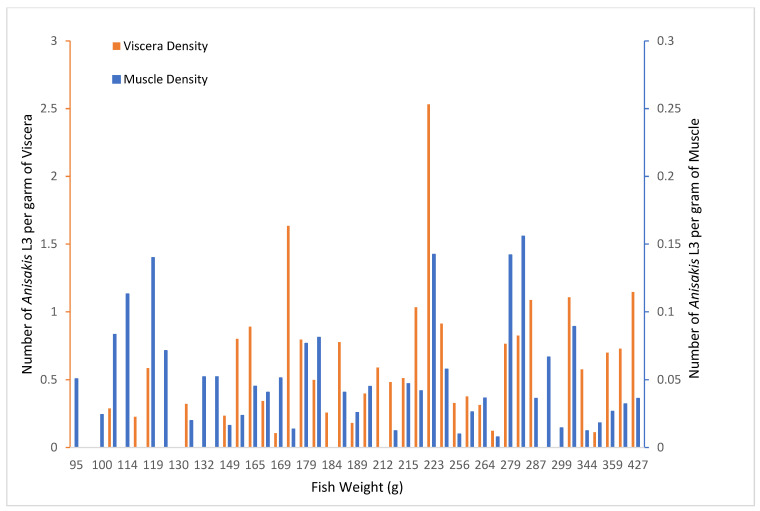
*Anisakis* spp. stage 3 larval density per gram of viscera and muscle in the second sample.

In the second sample, a significantly greater number of larvae were found in the viscera and the muscles of the heavier fish than in the lighter fish (Mann–Whitney U test, z = −3.55, and *p* < 0.01). The viscera density values were also significantly higher in the heavier fish (Mann–Whitney U test, z = −2.46, and *p* < 0.02); however, the muscle density values were not significantly different between the lighter and heavier fish (Mann–Whitney U test, z = −0.07, and *p* = 0.95) (Figure 2).

The larval burden results were as follows: mean abundance of 10.5, mean viscera larval abundance of 6.6, and mean muscle larval abundance of 4.31 (Table 2).

Several host–parasite parameters were analyzed statistically, such as fish length, weight, and condition factor with parasite abundance and density in the viscera and muscles using the Spearman correlation coefficient (ρ).

The larval abundance in the viscera was significantly correlated with the larval abundance in the muscle (Spearman correlation coefficient: ρ = 0.44, *p* < 0.01). However, there was no significant correlation between the density of the worms in the viscera and the density of those in the muscle (ρ = 0.21, *p* = 0.18) at *p* < 0.05.

Several host–parasite parameters were analyzed statistically, such as fish length, weight and condition factor with parasite abundance and density in the viscera and in the muscles using the Spearman correlation coefficient.

Fish length was significantly and positively correlated with the larval abundance per fish (Spearman correlation coefficient: ρ = 0.64, *p* < 0.01), the larval abundance in the viscera (ρ = 0.68, *p* < 0.01) and the larval abundance in the muscle (ρ = 0.39, *p* < 0.01). This was also the case with the density of parasites in the viscera (ρ = 0.49, *p* < 0.01). No significant correlation was found with the muscle density (ρ = 0.0, *p* = 0.99).

Fish weight was significantly and positively correlated with the larval abundance per fish (Spearman correlation coefficient: ρ = 0.63, *p* < 0.01), the larval abundance in the viscera (ρ = 0.67, *p* < 0.01), and the larval abundance in the muscle (ρ = 0.38, *p* = 0.01). This was also true of the density of parasites in the viscera (ρ = 0.45, *p* < 0.01) (Figure 2). No significant correlation was found with the muscle density (ρ = −0.02, *p* = 0.88) (Figure 2).

There was no significant correlation between fish condition and larval abundance per fish, viscera or muscle abundance, and the density of worms on the viscera or in muscle (ρ = −0.2 to 0.1, *p* > 0.38).

It is worth noting that the infection level of *Anisakis* spp. larvae detected in the first sample was lower than the values reported for *Anisakis* spp. in the same host from the same Atlantic area [21,22,35,36], but that reported in the second sample is very high. As regards the northern stock, Caballos-Mendiola et al. [33] found a very high prevalence (100%) of *A. simplex* s.s. in specimens caught on the Little Sole Bank (Northeast Atlantic). However, lower levels of infection have been reported in hake from the Mediterranean Sea [18,30], whereas higher levels of *A. pegreffii* infection have been recorded from the Adriatic and Ionian Seas when compared to specimens of a similar length from the Western Mediterranean [34].

Differences in parasite burden have been related to two drivers of infection, fish size and geographical area, especially in relation to its ecological conditions and the presence of suitable hosts for the life cycle of the *Anisakis* spp. [34]. Therefore, data from this study appear to agree with the existing hypothesis. The lower prevalence values observed in the first samples at the visceral level, in particular, during the present study may be due to the smaller length range (mean length 28 cm, range 24–33 cm) of the specimens compared to those examined in the second sample (mean length 31.6 cm, range 25–38 cm) and also in other cited studies, suggesting that these hosts likely have a younger age. For example, the length of the specimens analyzed is shorter than the mean TL (35 cm) reported for the specimens examined from off Portugal in Pascual et al. [22], where lower infection rates were found in smaller individuals. Higher infection rates, generally observed in larger and older fish [34], have been attributed to the cumulative effect of repeated parasite infections [38,39]. In addition, the different techniques used may have influenced the total number of larvae recovered, thus limiting comparison. As regards the present study, the utilization of compression and visual inspection (although on very thin muscle slices) used for our first sample, rather than more sensitive techniques, such as artificial digestion or the UV press method used for our second sample [40,41,42], might have influenced the negative results found at the muscle level. Moreover, different infection levels may be seen during different seasons throughout the year. Our first sample set was taken in autumn, when lower values were also noticed by Debenedetti et al. [40], compared to the spring values. Our second sample set, also taken during spring, also followed this pattern.

Although a significantly higher number of larvae were found in the viscera and the muscle of larger fish compared to the smaller fish in the second sample, the muscle density values were not significantly different between small and large fish. Larger fish will have a correspondingly greater muscle mass, causing a relative decrease in density of parasite larvae. It is also possible that older fish have developed a level of resistance to *Anisakis* infection, slowing down the migration of larvae from the viscera to the muscles. Strømnes and Andersen speculated that an increasing immune response may play a role in a seasonal drop observed in *A. simplex* abundance in fish species from Norwegian waters [43]. These factors may result in the density of larvae per gram of muscle remaining relatively constant as the fish increase in size.

The morphological identification used to separate *A. simplex* from *A. pegreffii*, suggested by Quiazon et al. [26], proved not to work well for our worms. Many *A. simplex* were initially wrongly identified as *A. pegreffii* using morphology but later confirmed as *A. simplex* by molecular characterization. These cases were probably young larvae that were not fully developed, and thus their ventricular length, the main feature proposed to separate them, was rather small, thus presenting dimensions similar to *A. pegreffi*. Quiazon et al. [26] reported values of 0.90–1.50 mm for *A. simplex* and 0.50–0.80 mm for *A. pegreffii*, and, consequently, if the larvae of *A. simplex* are not fully developed, they can easily be mistaken for *A. pegreffii*. This is supported by very recent work comparing morphological and molecular identification, where none of the studied morphological parameters could individually be used to discriminate between *A. simplex* s.s. and *A. pegreffii* based on morphometric measurements [44].

As regards molecular identification, both *A. simplex* and *A. pegreffii* were detected after database sequences comparison. In particular, the comparison of our 16 cox2 sequences (from *n* = 16 worms, 34.5% of the total worms collected in the first sample) with those deposited in GenBank allowed the identification of *A. simplex* in most cases (almost 70%) and of *A. pegreffii* in a smaller number of cases (about 30%). In fact, 11 sequences (Accession numbers MW073756-MW073766) resulted in an identification percentage ranging from 100 to 98.7% with sequences deposited as *A. simplex* (most frequent records: MN961158-59-62-71-72; GQ338428-30-33; KC810002-04; KX158869), whereas 5 sequences (Accession numbers MW073767-MW073771) gave an identification percentage ranging from 100 to 99.3% with sequences deposited as *A. pegreffii* (most frequent records: MN747366-367-372-401-404-410-426; MN961160-65; MN624202-211-413-414-415420-424). The results were confirmed by ITS sequences (sequences submitted to Genbank, access numbers OM418763-OM418769). These two species are known to occur in the European hake [21,22,34,36]: *A. simplex* s.s. is mainly distributed in the North East Atlantic Ocean [45] and off the Iberian Peninsula in mixed infection patterns of *A. simplex* s.s. (68.2% of the identified larvae), *A. pegreffii* (30.3%), and an F1 hybrid (1.5%), which has been reported [22]. The ratio between *A. simplex* s.s. and *A. pegreffii* found in this latter study [22] is similar to the ratio found in the present work. Unfortunately, the use of the cox2 gene and ITS in the present work does not allow the identification of hybrids; nevertheless, in the study by Pascual et al. [22], these were found only very infrequently, so it is not surprising that we did not find them in our sample. As already observed by Cipriani et al. [21], coinfection of *A. simplex* and *A. pegreffii* in the same host has been found in hake from the FAO 27 area. The presence of these species of *Anisakis* in our hake material is relevant from a public health point of view as, among the nine *Anisakis* species that have so far been described and genetically characterized [2,46], both *A. simplex* s.s. and *A. pegreffii* have been recognized as zoonotic species [47].

### 3.2. Revision of Human Anisakiosis Cases

In Portugal, data on human anisakiosis are scarce, and there are no official reports. Both Ramos [48] and Eiras [19] declared that, to the best of their knowledge, there were no notified cases of human infections in Portugal at the time of their articles’ publication. This was confirmed by the results of the present literature review, as the three cases, which we found and are discussed below, were all published very recently.

The first case occurred in a young man (32 years old) in Lisbon during 2017, who was admitted to the hospital with severe epigastric pain and was reported as having eaten sushi. A larva identified as *Anisakis* sp. was found by gastroscopy and removed, resulting in immediate recovery [49]. In the same year, an unusual hyperinfection was observed in a 43-year-old woman in Lisbon, who was admitted to a hospital emergency department following the sudden onset of severe symptoms the day after consuming grilled scabbard fish. Over 140 anisakid larvae were found in the woman’s stomach and removed by gastroscopy, again resulting in a rapid resolution of the symptoms. The parasites were identified as *A. simplex* s. l. [18]. Another case from 2018 was described in a 65-year-old woman presenting severe epigastric pain after eating raw fish. Endoscopy revealed the presence of six *Anisakis* larvae which were removed [50]. Moreover, an older case of eosinophilic esophagitis associated with recurrent urticaria, which was attributed to anisakiosis in a patient from Switzerland, was believed to have been acquired during a holiday in Portugal in 2000 [51]. In addition, in 2003, a study investigating specific antibodies to *A. simplex* amongst seafood consumers in the Algarve (southern Portugal), using a skin prick test (SPT), RAST, and assessment of total IgE and specific IgE, found eight individuals, of 100 analyzed, exhibiting specific antibodies to *A. simplex* in their blood. The authors concluded that their results indicated the probable existence, within the Portuguese population, of people that had been in contact with the parasite without becoming ill [52].

The current incidence of human cases of anisakiosis in Europe is unclear, but it appears to be fewer than 20 cases per country per year [53]. A total of 236 cases of anisakiosis were reported in a recent review of EU foodborne nematodiosis [54], but no data are available in the most recent EU summary report on zoonoses [55], as already observed [4]. As regards single Southern European countries, in France, a total of 37 cases were found between 2010 and 2014 [56], whereas, in Italy, between 1996 and 2018, 73 cases were determined [4]. In another retrospective study of Hospital Discharge Records (HDRs), also in Italy, 370 HDRs reporting the code for anisakiosis were retrieved between 2005 and 2015, especially from coastal territories of central and southern regions [57].

Unfortunately, in the published Portuguese cases of anisakiosis reviewed herein, the fish involved were never identified at the species level. The human case, with an unusually high number (140) of larvae found in the patient’s stomach [18], was attributed to the consumption of grilled scabbard fish, highlighting the importance of ensuring thorough cooking, even of the internal parts of the fish, which are the most heavily infected. In fact, in Portugal, it is very common to eat grilled fish that may not reach adequate temperatures for parasite devitalization in the deepest muscles (authors’ note). The implicated scabbard fish may indicate either or both *Lepidopus* spp. and *Aphanopus carbo*, species for which high prevalence values of *Anisakis* spp. are reported [58,59,60,61]. The other two cases were attributed to sushi [54] and generically to raw fish [55].

The potential risk represented by undercooked hake has recently been highlighted in a paper describing three cases of anisakiosis in Barcelona (Spain), after the consumption of fresh undercooked hake at home. During gastroscopy 12, 5, and 1 nematode larvae were detected in the three patients, removed, and later molecularly identified as *A. simplex* s.s. [17]. Furthermore, over 200 larvae were found in the stomach of another Spanish patient after the consumption of fried hake and fish ova [16]. The consumption of hake is also reported as having triggered a number of cases of allergic anisakiosis in Spain [17].

These cases confirm that in Europe the consumption of raw or undercooked fish has become a trend. If not adequately treated, these food-fishes are at risk of transmitting anisakiosis [2]. Such a risk is currently managed thanks to European legislation, which requires a preventive treatment of freezing in the case of fishery products to be consumed raw or almost raw and of smoked or marinated fishery products that undergo processing that is insufficient to kill and make safe any nematode larvae (Reg. EU 1276/2011) (Commission Regulation (EU), 2011) [23]. Moreover, in countries where this consumption is traditional, such as Spain, Italy, and the Netherlands, specific national regulations existed before European Regulations were in place [59]. In Portugal, however, national laws with more specific control measures beyond European legislation do not exist. Therefore, in addition to the obligations of Food Business Operators (FBOs) and the activities of official authorities, consumer education plays a pivotal role in reducing the transmission risk [56]. In addition, anisakid nematodes also negatively impact fish quality, and fish products containing visible live or dead larvae represent a risk according to Regulation (EC) No178/2002 [62]. Considering that European hake is a high-value commercial species in Europe [21] and that fisheries of the North East Atlantic supply valued products to regional European markets, in particular to southern European countries with high per capita fish consumption [22], the level of infection may influence the economic value.

Recent risk assessment studies made by Bao et al. stress that anisakiosis is an underdiagnosed zoonosis, and that the estimate of 500 cases per year in Europe is a strongly underestimated number [15]. In order to overcome this situation, the same authors also indicate the urgent need for educational campaigns to change consumer habits [15]. A quantitative risk assessment (QRA) for the Portuguese population, similar to that carried out by Bao et al. in Spain [15], would be very useful to get a more accurate picture of the risk posed to the general population from fish infected with *Anisakis* spp. The parasite burdens in hake detected in our study would be a key variable in such a QRA. Another relevant study was carried out by Caldeira, Alves, and Santos in 2021, which evaluated the notification of *Anisakis* spp., reported on the Rapid Alert System for Food and Feed (RASFF) portal between 1979 and 2019, where it was observed that, although the first notification of *Anisakis* spp. occurred in 2001, the largest number of cases occurred between 2011 and 2019 [11]. The main fish notified were mackerel, hake, and monkfish. This significant increase in notifications of *Anisakis* in recent years reinforces the need for the standardization of methodologies for detecting parasites using more modern techniques, as fishery products with parasites are still recurrent in the European market. Consumers play an important role in the seafood chain, and it is necessary to promote continuing education so that citizens can actively participate in the management of risks related to the ingestion of fish associated with *Anisakis* spp. larvae.

## 4. Conclusions

Although the consumption of raw fish, including hake, is traditionally unusual in Portugal, the increasing popularity of uncooked or lightly cooked seafood such as sushi may change culinary habits, making cases of human anisakiosis more common. Moreover, grilled fish prepared using a cooking method that does not reliably inactivate larvae is typically and frequently consumed in Portugal (authors’ note). In addition, the allergenic potential of dead *Anisakis* spp. larvae should be taken into account, especially considering the Portuguese habit of eating hake gonads, a typical site of *Anisakis* spp. larvae.

The high infection levels of *Anisakis* spp. larvae in hake, with 96% prevalence and more than 0.05 worms per gram of muscle, make this fish species particularly likely, if ingested raw, to infect humans and result in anisakiosis. Portugal should regulate the consumption of raw fish more tightly, with its own legislation, in order to prevent cases of anisakiosis. Additionally, a public site, where all notified cases could be published periodically, would be useful in order to better evaluate the national situation.

Unfortunately, in Portugal, parasites collected from the patients in the cases reviewed were not identified at the molecular level, which is an essential requirement for epidemiological surveys. Thus, an integration of data from fishermen, biologists, veterinarians, and physicians should be sought, pursuing a One Health approach, in order to provide policy makers with relevant and updated information for an appropriate, risk-based approach for seafood safety.

## Figures and Tables

**Table 1:** Summary of the host and parasite parameters determined for each sample.

Parameters	Sample 1	Sample 2
	(*n* = 30 hakes)	(*n* = 45 hakes)
Host		
Length (cm)	Mean ± SD (range)	Mean ± SD (range)
Weight (g)	Mean ± SD (range)	Mean ± SD (range)
Condition factor	-	Mean ± SD (range)
Parasites		
Prevalence (%)	Yes	Yes
Intensity	Mean ± SD (range)	Mean ± SD (range)
Abundance	Mean ± SD (range)	Mean ± SD (range)
Visceral abundance	Mean ± SD (range)	Mean ± SD (range)
Muscle abundance	Mean ± SD (range)	Mean ± SD (range)
Density (muscle)	No	Mean ± SD (range)
Density (viscera)	No	Mean ± SD (range)
Percentage of worms	Yes	No
found in each organ		

**Table 2:** Epidemiological parameters for each hake sample collected in the North Atlantic, off the Portuguese coast.

Epidemiological Parameters	Sample 1	Sample 2
Prevalence (95% CI)	76.7% (61.5–91.8%)	95.6% (89.5–100%)
Total n. of worms	46	473
N. worms in viscera	46	279
N. worms in muscle	0	194
Intensity (mean ± SD (range)	2.0 ± 1.2 (1–5)	11.0 ± 9.6 (1–41)
Abundance (mean ± SD (range)	1.6 ± 1.4 (0–5)	10.5 ± 9.7 (0–41)
Viscera abundance (mean ± SD (range)	1.6 ± 1.4 (0–5)	6.6 ± 7.1 (0–34)
Muscle abundance (mean ± SD (range)	0	4.3 ± 4.4 (0–20)
Density in muscle (mean ± SD (range)	-	0.05 ± 0.04 (0–0.16)
Density in viscera (mean ± SD (range)	-	0.54 ± 0.50 (0–2.53)
